# Female Fertility and Environmental Pollution

**DOI:** 10.3390/ijerph17238802

**Published:** 2020-11-26

**Authors:** Rita Canipari, Lucia De Santis, Sandra Cecconi

**Affiliations:** 1 DAHFMO, Unit of Histology and Medical Embryology, Sapienza, University of Rome, 00161 Rome, Italy; rita.canipari@uniroma1.it; 2 San Raffaele Scientific Institute, IRCCS H.S.Raffaele, 20132 Milano, Italy; desantis.lucia@hsr.it; 3 Dipartimento di Medicina Clinica, Sanità Pubblica, Scienze della Vita e dell’Ambiente, Università degli Studi dell’Aquila, 67100 L’Aquila, Italy

**Keywords:** ovary, hormones, endocrine disruptors, environmental pollution, heavy metals, female reproduction

## Abstract

A realistic picture of our world shows that it is heavily polluted everywhere. Coastal regions and oceans are polluted by farm fertilizer, manure runoff, sewage and industrial discharges, and large isles of waste plastic are floating around, impacting sea life. Terrestrial ecosystems are contaminated by heavy metals and organic chemicals that can be taken up by and accumulate in crop plants, and water tables are heavily contaminated by untreated industrial discharges. As deadly particulates can drift far, poor air quality has become a significant global problem and one that is not exclusive to major industrialized cities. The consequences are a dramatic impairment of our ecosystem and biodiversity and increases in degenerative or man-made diseases. In this respect, it has been demonstrated that environmental pollution impairs fertility in all mammalian species. The worst consequences are observed for females since the number of germ cells present in the ovary is fixed during fetal life, and the cells are not renewable. This means that any pollutant affecting hormonal homeostasis and/or the reproductive apparatus inevitably harms reproductive performance. This decline will have important social and economic consequences that can no longer be overlooked.

## 1. Introduction

Environmental pollution, which exerts potentially harmful effects on earth and atmospheric ecosystems, is caused by the presence of chemical, biological and physical substances [1,2]. It is a global problem shared by all developed and developing countries, but measures to prevent it are considered too costly. However, reduced environmental quality has long-term socioeconomic consequences: people are exposed to too many environmental toxicants, and their overall health conditions may worsen due to the synergistic and still-unknown effects of these factors on human health.

One of the most important and underestimated negative consequences is infertility, generally defined as “a disease characterized by the failure to establish a clinical pregnancy after 12 months of regular and unprotected sexual intercourse” [3]. It affects about 10–15% of couples aged 20–45 and affects women in 50% of cases. The most common direct or indirect causes of female infertility are advanced age, endocrine problems and damage to reproductive apparatus (vaginal, cervical, uterine, tubal and pelvic-peritoneal diseases). Premature ovarian insufficiency (POI), endometriosis and polycystic ovarian syndrome (PCOS) or sexually transmitted diseases have widely recognized roles in fertility failure [4], although approximately 15–30% of cases remain unexplained [5].

In the last few decades, increased age of first pregnancy can be considered the first cause of female infertility. Beside this, also the increasing incidence of cancer, the adoption of unhealthy lifestyles and exposure to environmental stressors play a negative role on it. However, while anti-cancer therapies are necessary, the negative effects of poor lifestyle choices (e.g., smoking, alcohol and drug abuse and excessive energy intake) and environmental pollution could be reasonably reduced, sometimes with easy changes of habits and better attention to our ecosystem.

In this review, we evaluate the effects and mechanisms of action of some of the most widely diffused contaminants—such as heavy metals (HMs), air pollutants and endocrine disruptors (EDs)—on female fertility. We also discuss the link between environmental pollution, climate and microbiome changes, because they can have surprising roles in reducing mammalian fertility.

## 2. How Environmental Pollution Affects Female Fertility

The impact of environmental pollutants has been extensively studied in recent years, and many papers have demonstrated how such chemicals impair human health (see for reviews: References [6,7,8]).

Environmental pollutants can permanently affect male reproductive potential [9], although these negative impacts can be attenuated by the presence of spermatogonial stem cells (0.03% of all germ cells) that in the seminiferous tubules is sufficient for maintaining fertility throughout the male lifespan. By contrast, in the mammalian ovary, the oocyte pool is fixed at birth, and the absence of stem cells hinders their replacement. Women produce a very small number (about 400) of potentially fertilizable oocytes from menarche to menopause, since a process of follicular degeneration (atresia) occurs throughout fetal and adult life, reducing the number of ovarian follicles by more than 99.9%. Some researchers claim that female fertility is not fixed after all, because their studies support the presence of stem cells in adult ovaries [10,11]. Recently, Wagner et al. [12] confirmed the absence of ovarian stem cells in samples of human ovarian cortices by using single-cell transcriptome and cell-surface-marker profiling, but this evidence was considered inconclusive by others. This is a puzzling issue, and there is no simple answer to it. Whatever the different opinions, all researchers agree that the production of fertilizable oocytes is a long and complex process dependent on strict collaboration between the germinal and somatic compartments of the follicle as well as on the coordinated interplay of several hormones. If this orchestration fails, there is no possibility to become pregnant.

## 3. Overview of Mammalian Oogenesis

A brief description of the key aspects of mammalian oogenesis and its hormonal regulation is useful to highlight that the complex molecular relationships among ovary, paracrine and endocrine factors regulate the capacity to produce a fertilizable oocyte, an event that can be considered an indicator of a woman’s health status.

**Figure 1:**
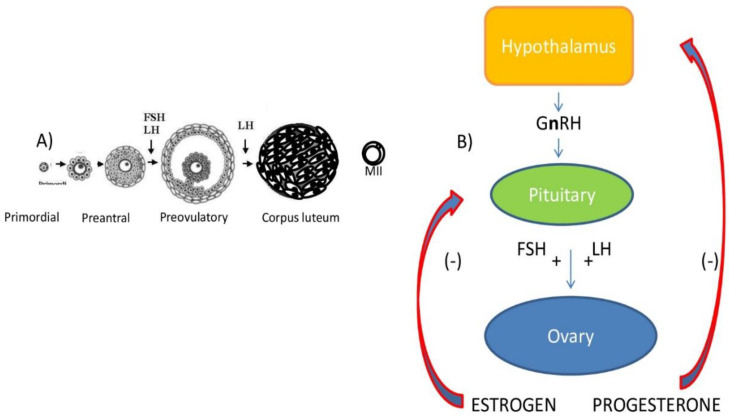
Ovarian follicle development and hypothalamic/pituitary/ovarian axis. (**A**) Schematic representation of mammalian follicle development from primordial stage to corpus luteum formation. The ovulated oocyte is arrested at metaphase II (MII) until fertilization. (**B**) GnRH (Gonadotropin releasing Hormone) stimulates FSH and LH release. When gonadotropins bind specific receptors present on ovarian somatic cells, they stimulate estrogen and progesterone production, which exert negative feedback on gonadotropin release.

The precursors of oocytes are the proliferating primordial germ cells (PGCs), which appear during fetal life, when they migrate towards the genital ridges, entering meiosis that is arrested at the diplotene stage of prophase I, commonly known as the germinal vesicle (GV) stage. The maintenance of this quiescent or resting stage lasts for months in rodents and years in humans [13], thereby exposing germ cells to all environmental toxicants from prenatal to adult life. The pool of primordial follicles, formed by the GV-arrested oocytes, surrounded by a single layer of flattened granulosa cells (GCs), is usually formed during the first few days after birth in rodents, and during fetal life in primates (Figure 1A).

**Figure 2:**
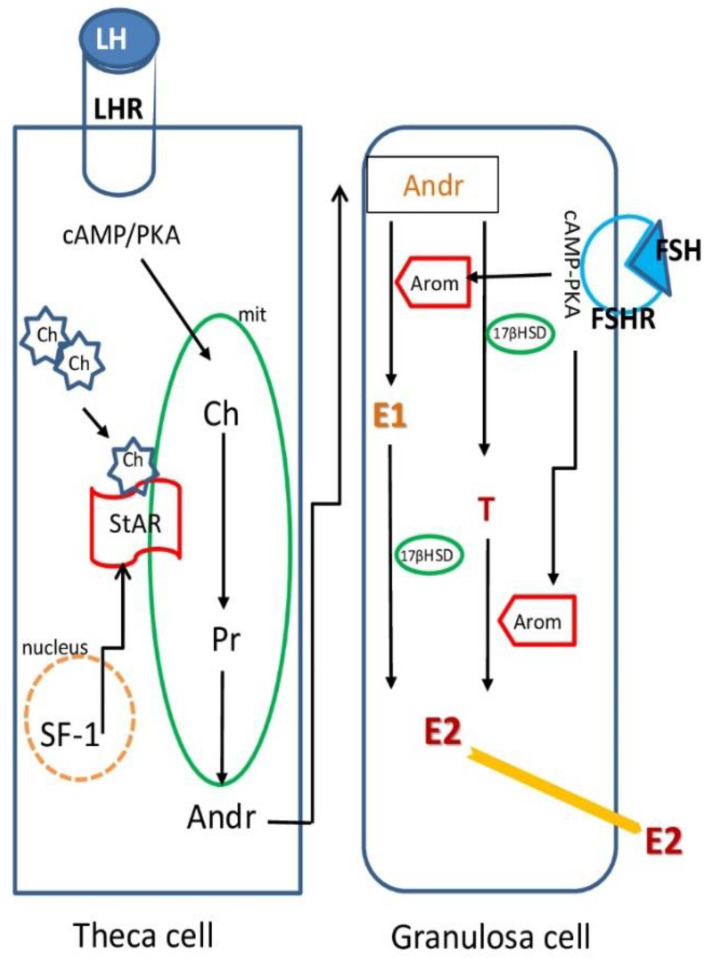
Two cell, two-gonadotropin theory. Ovarian steroids are synthesized from cholesterol (CH) via the cooperation of theca and granulosa cells. In theca cells, LH and its receptor (LHR) stimulate androgen synthesis via Gαs-mediated increases in cAMP that, in turn, activates PKA. This kinase can increase steroidogenic acute regulatory protein (StAR) expression and activity in collaboration with the orphan nuclear receptor Steroidogenic factor-1 (SF-1) an), which acts as a global regulator of steroidogenesis. StAR moves cholesterol (Ch) into the mitochondria (mit), where it is converted to progesterone (Pr) and then androstenedione (Andr). In granulosa cells, FSH and FSHR stimulate the activity of 17βhydroxysteroid dehydrogenase (17βHSD) and aromatase, which produce estron (E1) and estradiol (E2) from Andr and T (testosterone).

The first wave of follicle recruitment consists of the transition from the resting to the growing phase (activation): this event is controlled by intraovarian factors, either activatory or inhibitory, that must be accurately balanced to avoid the premature exhaustion of the pool of resting follicles and, therefore, reproductive potential [14]. GC proliferation and oocyte growth characterize preantral follicle development. At this point, GCs express receptors for follicle-stimulating hormone (FSHR). Despite the fact that early folliculogenesis is being considered a gonadotropin-independent phase, FSH stimulates follicular growth, as evidenced by increased aromatase-Cyp19a1 expression and estradiol (E_2_) production, which, in turn, regulates GC proliferation. Preantral follicles are also responsive to various members of the transforming growth factors (TGF) family (such as TGF-β, bone morphogenetic proteins and growth and differentiation factor-9), as well as to androgens, insulin and insulin-like growth factor-1. The formation of theca cell (TC) layers from the ovarian stroma occurs in a gonadotropin-independent manner and provides the follicles with luteinizing hormone (LH) receptors (LHR). The complex pathways stimulated by gonadotropins are represented in a simplified form in Figure 1B and Figure 2. Starting from cholesterol, LH regulates the production of androgens that are converted to E_2_ in GCs. Estrogens also regulate FSHR activation and the induction of LHR in the GCs [15].

During the growth phase, the oocyte also secretes the zona pellucida, a glycoproteic matrix surrounding the oocyte responsible for the polyspermy block at fertilization. To coordinate the development of follicles, either the gap junction-dependent transfer of nutritive/regulative molecules or the release of stage-specific soluble paracrine factors is required. This fine orchestration drives not only somatic cell differentiation and steroidogenesis but also oocyte maturation [16,17]. The pulsatile secretion of FSH stimulates the transition to antral phase, which is marked by the appearance of an antral cavity filled with follicular fluid (FF) and by the oocyte-dependent differentiation of the cells surrounding the oocyte, the cumulus cells (CCs) and mural GCs (MGCs) lining the antrum. FSH selects a cohort of antral follicles, and among them, one (human) or more (polyovulatory species), characterized by a higher mitotic rate of GCs and elevated E_2_ production in comparison to others, become the dominant follicles. The E_2_-dependent negative feedback on FSH secretion causes the atresia of the less-mature follicles. The preovulatory follicle and its enclosed GV-arrested oocyte have at this point reached their maximum sizes (in humans: follicle > 10 mm; oocyte: about 120 µm in diameter) [18] and are both ready to respond to the LH surge that triggers meiotic maturation and ovulation. It is remarkable that throughout the female reproductive life, all the different follicle types are simultaneously present in the ovary.

Meiotic resumption is an elegant process that requires a complex interplay of several hormones (FSH, LH and E_2_) and signaling pathways [19,20,21]. The LH signal is transmitted from MGCs to oocytes via the production of epidermal growth factor (EGF)-like factors, and the subsequent activation of MPF (Mitosis Promoting Factor) and MAPK (Mitogen-Activated Protein Kinase) signaling culminates with the formation of the first meiotic spindle together with the extrusion of the first polar body (PBI), containing the redundant genetic material, followed by the arrest of division at metaphase II (MII). Both nuclear and cytoplasmic maturation (about 24 h in humans) are required to produce a viable embryo. Following the rupture of the follicular wall, the ovulated oocyte is released into the fallopian tubes, and LH induces changes that convert the follicle remnants into an endocrine structure known as the corpus luteum (CL) (Figure 1A). The release of progesterone (Pr) is necessary to prepare the endometrium for implantation [22] and to slow the growth of other follicles [23]. The second meiosis is completed only after fertilization and is evidenced by extrusion of PBII and the formation of male and female pronuclei (2PN).

### 3.1. Toxicity of Heavy Metals (HMs)

HMs are a serious health problem due to their accumulation in soil, water and the food chain and their resistance to decomposition in natural conditions. Not all the metals are obligatorily toxic. Some, such as copper, chromium, manganese and zinc, are essential at very low concentrations but toxic at higher ones; others, such as cadmium (Cd), mercury (Hg) and lead (Pb), do not have metabolic roles and are toxic at all concentrations. The predominant source resulting in measurable human exposure to HMs is the consumption of contaminated drinking water, which often contains a mixture of arsenic, Cd, nickel (Ni), Hg, chromium (Cr), zinc (Zn) and Pb [24,25]. Moreover, sea and river contamination cause the accumulation of HMs in many fish. Although fish consumption is recommended because of fish’s high content of omega-3 polyunsaturated fatty acids, e.g., eicosapentaenoic acid (EPA) and docosahexaenoic acid (DHA) [26,27], some concern has been raised about the presence of high levels of MeHg in fish at higher trophic levels, such as walleye, pike, swordfish, tuna and shark, because of bioaccumulation and biomagnification of Hg. People who frequently consume these fish species are regarded as being at relatively high health risk [28]. Other important sources of HMs are cigarette smoking, occupational exposure from various industrial processes known to utilize these metals, rechargeable Ni-Cd batteries, jewelry, solders, color pigments and alloys.

**Figure 3:**
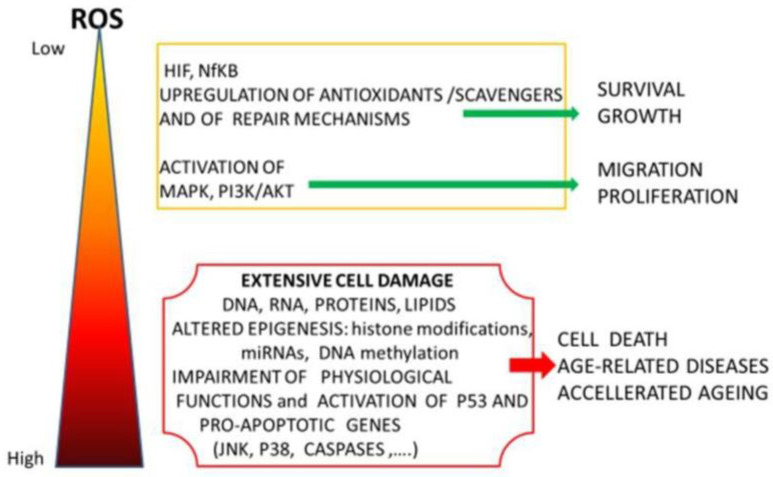
Schematic representation of cellular effects of various reactive oxygen species (ROS) levels. While low levels stimulate cell recovery/survival, high levels alter epigenetic mechanisms and induce degenerative processes or apoptosis via activation of p53 and pro-apoptotic genes. HIF (Hypoxia inducible factor) and NfKB (nuclear factor kappa light-chain-enhancer of activated B cells) are both involved in inflammation response, MAPK and PI3K/AKT (phosphatidylinositol 3-kinases/protein kinase B). are major regulators of cell cycle and survival and JNK (c-Jun N-terminal kinase) and P38 are key mediators of oxidative stress often associated with apoptosis and increased caspase activity.

In addition to the well-known capacity to induce cardiovascular, renal and neuronal damage and increase the risk of cancer and diabetes, in recent decades, there has been increasing interest in the possible detrimental effects of HMs on human fertility. Even though HMs can affect fertility in both sexes, females are more affected because of the fixed and non-renewable pool of germ cells in the ovary. Deleterious effects can be observed at several stages of reproductive life in females, from fetal life to puberty and maturity [29,30]. Data indicate that HMs can influence gene expression by modulating epigenetic mechanisms and the expression of non-coding RNAs, especially microRNAs. Moreover, chronic exposure causes steroidogenic dysfunction, fetal abnormalities and embryotoxicity because many HMs, such as Cd and Ni, act as endocrine disruptors (EDs), capable of manipulating the production and activity of hormones and their receptors [31]. HMs have also been reported to enhance oxidative stress (OS), thereby affecting a range of physiological processes involved in hormonal homeostasis and germ cell and embryo quality. All these negative effects may ultimately contribute to infertility [32]. It has been shown that involuntary exposure to HMs during pregnancy is directly related to preterm birth due to excess reactive oxygen species (ROS), particularly the significant increase in OS in the trophoblastic placental tissue [33] (Figure 3).

Placenta serves as an interface between maternal and fetal circulation and plays an important role as a mediator of nutrient transport and as a barrier for toxic substances. However, the human placenta does not block the passage of all toxic elements and non-essential metals can cross this barrier due to their size and charge, similar to those of essential metals, posing a potential risk to human fetus [34,35]. Some studies have also investigated correlations between heavy metals concentration in the placenta and fetal growth and development, which may then lead to severe fetal damage [36,37]. Therefore, the placenta has been identified as an indicator of fetal exposure to toxic metals [38,39].

The effects of the most-diffused HMs on female reproduction are reported below.

#### 3.1.1. Lead (Pb)

Lead’s toxicity and effects on reproductive performance have been suspected since antiquity [40]. However, the first data documenting adverse effects on pregnancy were published in 1860 [41] in France.

Lead levels in blood are classified into four different groups, as normal (<4 μg/dL), mild (5–9 μg/dL), moderate (10–14 μg/dL) and high (15–20 μg/dL). Any amount exceeding these levels is classed as severe. Continuous exposure to Pb over a lifetime or in an adolescent monkey, with approximately 35 μg/dL in the blood, caused a Pb-dependent decreased production of circulating gonadotropins and E_2_, leading to menstrual irregularities, spontaneous abortion and fetal anomalies [42], while gametotoxicity and interference with the normally occurring increase in steroid hormones during implantation have been described in Pb-treated mice [43,44]. A relevant teratogenic effect occurs in mouse female fetuses, which, later, in adult life, show a reduced number of PGCs and of implanted embryos, as well as high preimplantation mortality [45,46].

However, in humans, the maternal effects of Pb toxicity are not so clear. While it has been reported that females who experienced lead intoxication during their childhood have a significantly high rate of spontaneous abortion [47,48], with a striking dose-response relation with blood lead levels, in other studies, no significant correlation has been shown between the pregnant woman who were exposed to lead intoxication and those who spontaneously aborted [48,49,50]. However, in these cases, these people had no history of lead exposure during their childhood, while a significantly higher proportion of spontaneous abortions or stillbirths was found in women who experienced Pb poisoning during childhood [51]. In fact, Pb can also be transferred during pregnancy to the fetus and during lactation to the child. It has been demonstrated that a high percentage of Pb can be accumulated in the human skeleton during childhood [52].

Moreover, during pregnancy, the high demand for calcium can lead to bone demineralization and the co-mobilization of Ca and stored Pb, which becomes the main source of fetal poisoning [49,53]. The impaired fertility of females born from mothers smoking during pregnancy confirms that the period of fetal ovary development is critical for fertility in adult life [54]. Therefore, even though a direct correlation between exposure to Pb and miscarriage is not so evident in humans, the possibility of reproductive defects after childhood Pb exposure cannot be ruled out.

#### 3.1.2. Cadmium (Cd)

Cadmium accumulates in the body over time, gradually increasing with years of exposure. It can be measured in the blood, urine, hair, nails and saliva: the tolerable standard limit in the saliva is 0.55 μg/L for humans [55].

This metal can be found in batteries (specifically, rechargeable Ni-Cd batteries) as well as in foods rich in fiber (e.g., vegetables, cereals, potatoes and spinach). However, the major routes of Cd exposure are the inhalation of fumes and dust, and active/passive cigarette smoke. In fact, when comparing smoking with nonsmoking females, higher concentrations of Cd were found in the smokers (median, 1.3 versus 0.32 μg Cd/L). Even though Cd retention can affect both sexes, it is commonly higher in women than in men. The cause of this distinction is the elevated gastrointestinal uptake of this HM caused by the depletion of iron stores, a situation common in women at fertile age, especially during pregnancy [56,57]. After menopause, blood Cd concentrations gradually become comparable to those of men, due to the reduced gastrointestinal absorption of Cd associated with improved iron status [58]. Thus, the maintenance of a correct level of iron in women is important for preventing/reducing the uptake of Cd [59]. It has been shown that in vitro, Cd at a concentration as low as 5 μM can interfere with the biosynthesis of Pr by decreasing the expression of enzymes important in steroidogenesis, such as P450 cholesterol side-chain cleavage (P450scc) and 3β-hydroxysteroid dehydrogenase (3β-HSD) in placental cells [60]. Moreover, it has been shown that Cd activates the estrogen receptor by binding to the hormone-binding domain of the receptor [61]. These results can explain the consequent delay in puberty/menarche, loss of pregnancy, menstrual disorders, hormonal impairments, premature births and reduced birth weights [62]. The possible mechanisms by which Cd affects steroidogenesis include interference with DNA-binding Zn-finger motifs through the exchange of Cd for Zn, and a role as an ED, able to mimic or hinder the activities of endogenous E_2_ [63].

#### 3.1.3. Mercury (Hg)

The World Health Organization (WHO) has estimated that the mean total blood Hg concentration for the general population is around 8 μg/L, but blood concentrations up to 200 μg/L can be detected in the case of high fish consumption. The main sources of Hg exposure are coal combustion, mining and the chemical industry [64]. Hg can be transformed into highly hazardous forms such as methylmercury (MeHg) and ethylmercury (EtHg) by microorganisms and bacteria [65,66]. These modified forms can accumulate in freshwaters, ecosystems and food chains, and fish represent the main source of Hg exposure for people and other living organisms. Hg can affect both male and female fertility, but studies on female fertility are, once again, relatively scarce. It has been shown that Hg influences the levels and function of E_2_, and that it can cross the placental membrane, thereby inducing spontaneous abortions, premature births and congenital defects [67]. Moreover, Hg exposure has been correlated with pathologies such as PCOS, endometriosis, premenstrual syndrome, dysmenorrhea, amenorrhea, breast disorders and abnormal lactation [68].

#### 3.1.4. Zinc (Zn), Cobalt (Co) and Nickel (Ni)

In the literature, conflicting results can be found for other, less-studied HMs. Elevated concentrations of Zn can cause defective embryogenesis to even having teratogenic and lethal effects [69], while nutritional deficiencies in the maternal diet can have effects on the growth of offspring [70]. The normal levels of Zn in the blood are 70–120 µg/dL, while values lower than 70 µg/dL are defined as zinc deficiency [71]. Maternal zinc deficiency may have adverse consequences for offspring, either as an acute effect during pregnancy or through their lifespan, by increasing their susceptibility to diseases as an adult. Suboptimal zinc consumption in humans was associated with increased premature births, low birthweights and increased congenital malformations, which are all possible acute effects of zinc deficiency [72,73,74]. In addition, Zn deficiency can negatively affect oocyte maturation, cumulus expansion and ovulation because meiotic arrest and cumulus expansion are two essential Zn-dependent ovarian processes [75].

Co is an essential oligoelement that is part of Vitamin B12. It is essential at low concentrations in humans for the formation of new red blood cells but is toxic at high concentrations [76]. Its daily intake ranges from 1.7 to 100 μg/day. Co mainly accumulates in the liver and kidneys, and the total levels in the body tend to be around 1.5 mg [77]. Co can mimic hypoxic conditions because it acts on the stabilization of hypoxia-inducible factor (HIF), which activates several responsive target genes involved in angiogenesis and the regulation of apoptosis/cell proliferation, thereby promoting cancer progression [76] (Figure 3). The continuous exposure of female mice to an average daily dose of 8–16 rad impaired reproductive performance, decreasing the number of offspring per litter [78], and in female mice exposed to 11.4 mg of Co/m^3^ daily for 13 weeks, a significant increase in the length of the estrous cycle was reported [79]. In humans, Co causes menstrual problems, altered sexual behavior, infertility, an altered onset of puberty, an altered length of pregnancy, lactation problems and altered menopause [80].

The effects of Ni exposure on female reproductive functions remain unclear, and information available from human or experimental studies is sparse, even though the embryotoxicity and carcinogenicity of Ni have been documented (for a review, see Reference [81]). In female rats, the 5-day administration of 40 mg/kg of body weight of NiSO_4_ inhibited ovulation and abolished P4 production in response to LH. Moreover, the administration of 40 mg/kg has been reported to disturb menstrual cycles, decrease the implantation of embryos and increase embryo resorption [82].

## 4. Air Pollution

Every day, people living in industrialized countries breathe and ingest a mix of particles and chemicals present in the air, many of which may also enter the food chain via the contamination of soil and water. The list includes particulate matter (PM; diameters: 10, 2.5–10 and 2.5 µm), ground-level ozone (O_3_), benzo(a)pyrene (BaP, the main marker of polycyclic aromatic hydrocarbon (PAH) presence), polychlorinated biphenyls (PCBs), sulfur dioxide (SO_2_), nitrogen dioxide (NO_2_), carbon monoxide (CO), organic compounds (organic solvents and dioxins) and HMs, all abundantly produced by transport and industries.

A key question is how unavoidable maternal exposure to contaminants during the pre- and peri-conceptional periods causes abnormalities in oocytes, embryos and/or fetuses and is able to hamper the safe delivery of a baby and his/her overall health and mental activity.

The main air contaminants appear to impair both animal and human gametogenesis and to lead to a drop in reproductive performance [83,84]. The main mechanisms by which they affect the ovary rely on their capacity to alter the endocrine system, to increase OS and inflammation and to activate specific targets able to stimulate inappropriate MAPK signaling [85,86,87,88] (Figure 3).

Several reports have claimed that women living in highly industrialized areas have fewer fertilizable oocytes due to a significant decrease in antral follicle numbers, to a lower fertility rate (number of live births per 1000 women) and to a higher implantation failure rate in comparison with controls [83,84]. Xue and Zhang [89] found that PM 2.5 impaired sperm and oocyte quality, decreasing fertility by 2% per 10 µg/m^3^ increment in these fine particles. Since the HMs (e.g., Pb), PHAs and PMs present in waste gas can have estrogenic or anti-estrogenic/androgenic activities [84], general imbalance in the endocrine system could be responsible for the impairment of gonadal steroidogenesis and, therefore, gametogenesis as a whole (Figure 1 and Figure 2). Such a negative role has been confirmed by Gaskins and collaborators [90], who showed that air pollution accelerates reproductive ageing by decreasing the ovarian reserve. Recently, Santi et al. [91] assessed the existence of a link between higher air pollution and reduced fertility by determining serum levels of AMH. This hormone is released by ovarian somatic cells and is used as a marker of a woman’s ovarian reserve, which represents the number of viable eggs the ovaries can produce. After measuring the daily levels of PM2.5–10 and NO_2_ in areas around Modena City, they measured serum AMH levels, finding a significant decrease in AMH in women living in the worst-polluted areas. Although the relationship between AMH levels and the chances of becoming pregnant naturally is still debated, these results support the idea that environmental factors can interfere with ovarian physiology. Experiments conducted with female mice exposed to diesel exhaust particles or PM2.5 before conception showed inner cell mass-trophectoderm differentiation arrest at the blastocyst stage, defective post-implantation embryonic development, decreased numbers of viable fetuses and higher rates of miscarriage [92]. Moreover, women exposed even for a short time to high levels of PM10 during the preconception period experienced early pregnancy loss after in vitro fertilization (IVF) at a higher frequency than controls. For this reason, it has been proposed that IVF procedures should be avoided when environmental stressors cannot be excluded during such a sensitive phase [93]. The negative impact on the fecundability rate has been confirmed by Slama et al. [94], who found that increases in PM2.5 and NO_2_ levels are associated with a significant decrease in fecundability, especially in the first month. Other reports have demonstrated that the link between the fertility rate decrease and traffic-related pollution is a function of residential exposure [95] as well of proximity (<200 m) to the main road [96]. More recently, Conforti et al. [84] analyzed data from the literature on the impact of the most common pollutants on IVF for women, finding that exposure to PM2.5 and PM2.5–10 reduced the conception rate (odds ratio (OR), 0.9; 95% confidence interval (CI), 0.82–0.99; per 8 µg/m^3^ increase), while SO_2_, CO and NO_2_ seemed to be more responsible for miscarriage and stillbirths. Additionally, the observation that O_3_ and PMs (especially sulfate compounds) significantly increase (+13%) the risk of miscarriage [97] should be further investigated. Even though the normal development of a fetus can be hampered at any time, Zhang et al. [98] reported that exposure to SO_2_ during the first trimester can affect the baby’s health.

During pregnancy, fetal growth is accompanied by morphological changes of the placenta such as extensive angiogenesis in uteroplacental and fetoplacental vasculatures as well as increases in uterine and umbilical blood flows [99]. These changes are essential for a correct fetal development [100,101,102]. Thus, factors that affect vascular development and function will have impacts on fetal growth, development and survival [101]. It has been shown that air pollution affects the functional morphology of mouse placenta [103] and the authors hypothesized that alterations in its functional morphology could at least contribute to the reduced fetal weights associated with exposure to air pollution. Recently, Segal and Giudice [104] proposed that reproductive endocrinologists and gynecologists should promote healthy pregnancies by educating fertile women to adopt safe lifestyles during the preconception period, including the indoor use of High Efficiency Particulate Air (HEPA) filters, and to avoid outdoor activities when the air quality is poor due to heavy traffic.

In this context, smoking habit represents a serious problem. About half of the exposure to benzene in the United States results from direct or indirect exposure to tobacco smoke (U.S. Department of Health & Human Services, 2019). A recent report by the American Society of Reproductive Medicine [105] indicates that in the USA, about 15% of adult women are smokers but, at the same time, often unfamiliar with the consequences of smoking on their reproductive apparatus. More importantly, it is generally accepted that people exposed to secondhand smoke can suffer the same health risks of smokers [106]. A cigarette contains approximately 600 ingredients and, when burned, creates more than 7000 chemicals, of which at least 70 are known to cause cancer [107]: nicotine, NO_2_, formaldehyde, CO, HMs, tar and benzene are some examples.

Benzene, one of the chemicals produced by cigarette smoke, has been measured in the FFs of women undergoing IVF, and when it was present at >0.54 ng/mL, women showed higher basal FSH levels and significant reductions of E_2_ and the numbers of oocytes retrieved, and embryos transferred [89]. Additionally, smoking stimulates follicular depletion, an increase in mean basal follicle FSH levels and the bringing forward of menopause by 3–4 years [108,109]. More recently, Furlong et al. [110] found that cigarette smoke induced ovarian dysfunction by dysregulating the expression of 152 miRNAs, five of which directly affect the MAPK pathway. It is noteworthy that the overexpression of the phosphorylated form of) MAPK is typical in ovarian cancers [111,112].

Finally, it is of interest to highlight the consequences on fertility of the use of sprayed pesticides. The dermal and inhalation routes of entry are typically the most common routes of farmers’ exposure [113], although people living in areas treated with pesticides can also be subjected to direct spray diffused from neighboring fields. Some mechanisms of action of these pesticides can be explained by using laboratory animals. An interesting example is that of mancozeb [87,114,115], a fungicide used for the control of fungal plant pathogens and widely used to protect vegetables (tomatoes and potatoes), fruit (grapevines, apples and bananas) and ginseng, as well as ornamental plants and golf courses. Since mancozeb is usually sprayed with aerial equipment, the general population can be easily exposed by inhalation and/or the ingestion of contaminated food. Despite its low acute toxicity, mancozeb impairs fertilization and embryo development in female mice exposed to high doses (500 mg/mL) during pregnancy and lactation [116]. In vitro, low doses of mancozeb (0.001–1 µg/mL) alter GC morphology [88,117] and mitochondrial metabolism [87]. Similar effects on fertilization have been observed for other sprayed pesticides [118].

## 5. EDs: Phthalates and Bisphenol A (BPA)

Chemical compounds such as Bisphenol A (BPA) and phthalates are widely used in many daily consumer products and have long been indicated as EDs. Phthalates are a large group of substances classified as short-chain or long-chain phthalates according to their low or high molecular weight [119]. They are mainly used as plasticizers in polyvinyl chloride (PVC) products and are produced by an esterification process with different substituents of phthalic anhydride. In addition, some phthalates (in particular, dibenzyl phthalate (DBzP), diethyl hexyl phthalate (DEHP) and dimethyl phthalate (DMPH)) are classified as highly toxic substances by REACH (Registration, Evaluation, Authorization and Restriction of Chemical substances) based on animal-reproduction studies [120].

BPA, a key monomer in the production of epoxy resins and the most common forms of polycarbonate, has detrimental effects on reproductive health. It is mainly used for plastic materials, and its derivatives have been on the market for over half a century, as it is an almost unbreakable material [121]. However, since these molecules do not establish stable and irreversible bonds with the materials in which they are embedded, they can leak from the plastic matrices and migrate into food or drink, especially if these are lipophilic, a process further accelerated by heating. For this reason, human exposure to these toxicants occurs mainly through the ingestion of contaminated water and food [122]. Both phthalates and BPA, through numerous mechanisms of hydrolysis, oxidation and conjugation with hydrophilic molecules, are therefore able to migrate in many organs and be excreted in the urine [120,123,124]. Considering metabolic and excretory pathways, phthalates and BPA have long been subjects of study for their possible deleterious effects on human reproduction, particularly on female fertility.

In rodents, DEHP seems to affect all steps of follicle development, from PGC formation to ovulation (Figure 1A). In fact, DEHP exposure during fetal life (10 mg/kg bw/d) induces a significant decrease in oocyte number, the dysregulation of meiotic progression and the anomalous activation and depletion of the pool of resting follicles by increasing ROS levels [125]. In the growing follicles, phthalates stimulate atresia by altering steroidogenesis and increasing OS; after the LH surge, they can impair oocyte meiotic maturation and ovulation. The negative impact on oocytes is likely mediated through the hyperactivation of E_2_-dependent genomic/non-genomic pathways in the surrounding somatic cells. Since estrogen receptor alpha (ERα) can interact with several enzymes inducing epigenetic changes (e.g., acetylases/deacetylases and methylases/demethylases), DEHP can induce epigenetic modifications that are potentially inherited by subsequent generations [125]. These toxic effects have been evaluated recently with experiments in which adult female mice have been chronically exposed to a mixture of phthalates and alkylphenols at environmentally relevant doses. The results clearly show that exposed mice have deregulated estrous cyclicity because of the altered expression of the enzymes involved in the synthesis of steroid hormones [126] (Figure 2).

The interference of BPA and phthalates with ovarian development can lead to varying degrees of infertility [127]. However, while high urinary levels of phthalate levels, especially MEHP (0.69 µg/L), have been associated with a significantly higher risk of implantation failure in IVF women, high BPA levels have been associated with a decrease in the antral follicle count and the number of oocytes, with possible links to endometriosis [128]. Numerous animal studies have shown that exposure to phthalates inhibits androgen production in males [129]. By contrast, BPA has a binding affinity for androgen (AR) and estrogen (ER) receptors, thus causing their dysregulation [130]. Human studies are complex, especially due to a series of confounding factors that often create bias in the setup of experiments. However, some evidence seems to refute the real presence of these compounds in the FFs at worrying doses [131], while other evidence appears to demonstrate a positive association between exposure and presence at the reproductive tract level [132]. In addition, widespread environmental pollutants, such as Bisphenol A, have been reported as potential contributors to the pathogenesis of polycystic ovarian syndrome (PCOS) [133,134,135].

Finally, it is apparently confirmed that there is a correlation between the geographical region of origin and/or residence and the presence of EDs in the FF recovered in IVF cycles, confirming the hypothesis of continuous exposure over time affecting the functionality of the reproductive tract [136].

Recent reports evidenced that dogs can be a useful model for studying phthalate effects, because exposed animals show impairment of sperm quality similar to that of humans exposed to this toxicant [137]. If confirmed, the identification of a “human-like” model could be an important opportunity for increasing our knowledge of toxicant effects also on females.

## 6. Thermal Stress

The rising external temperatures and extreme weather will precipitate, probably during this century, not only a significant increase in sea and CO_2_ levels but also a wide range of health issues, including the drastic impairment of the reproductive efficiency of plants and animals [138,139]. Even if mammalian species can maintain constant body temperatures, elevated external temperatures significantly perturb oocyte and embryo production [140]. Indeed, steroid production is strongly affected by seasonality [98,141,142], and during summer, the Pr level in FFs and blood is significantly decreased, which is responsible for a higher incidence of preimplantation embryo death [143]. Many reports have demonstrated that somatic cells in the ovaries of farm animals exposed to elevated thermal stress have high levels of DNA damage and apoptosis, and the upregulation of antioxidant genes has been described to occur in porcine [144] and mouse [145] GCs exposed to hot temperatures (28–40 °C for 2 weeks).

## 7. Conclusions

A woman’s fertility is dependent on the timely and appropriate orchestration of ovarian and hormonal functions, and any dysregulation of the signaling pathways involved in physiological oocyte and/or embryo development increases the difficulties in becoming pregnant. Unfortunately, studies on the effects of pollutants on female reproduction mostly describe the effects of a single agent, while women, as a general population, are exposed to a combination of several harmful toxicants daily. Therefore, even though the concentration of a single agent may be low, the synergistic action of different pollutants, together with predisposing factors, can increase the risk of developing diseases.

The link between environmental pollution and female fertility will be better assessed in the next years, maybe by also considering other parameters beside fertility rate, such as the frequency of congenital anomalies [146]. In fact, recent reports highlighted that environmental pollution and climate change are significantly increasing the risk of birth defects, especially after prolonged exposure [147,148]. Indeed, it has been proposed that timing and duration of maternal exposure to a complex mixture of environmental chemicals impairs the fetal neuroendocrine system in a sex-specific manner, as expression of some genes involved in neuroendocrine development is altered in male fetus in comparison with control [149].

Therefore, even if the consequences of environmental pollution for human health are still a matter of debate and deserve further study, the data reported in this review support the idea that a safer ecosystem can contribute significantly to reproductive health.
